# Preparation and characterization of nanomuscovite by intercalation method for adsorption of heavy metals from polluted water

**DOI:** 10.1007/s10653-023-01545-4

**Published:** 2023-04-19

**Authors:** M. Nageeb Rashed, A. E Arifien, F. A. El-Dowy

**Affiliations:** Chemistry Department, Faculty of Science, Aswan University, Qism Aswan, Egypt

**Keywords:** Adsorption, Heavy metals, Nanomuscovite, Pollution, Wastewater

## Abstract

In this study, nanomuscovite adsorbents were prepared by intercalation with various organic intercalates (DTAB-TTAB-DTPA-PA-PN) and used to remove Cd^2+^ and Pb^2+^ from polluted water. The best nanomuscovite was prepared using DTPA and muscovite (Muc/DTPA) and characterized by XRD, TEM, EDX, FTIR, and BET surface area. The developed nanoadsorbent was used to remove Cd^2+^ and Pb^2+^ from polluted water. The effect of various factors, including contact time, adsorbent dosage, solution pH, and temperature, was investigated. The results reveal that the maximum adsorption of Cd^2+^ and Pb^2+^ was 91.5% and 97%, respectively, at the initial metal concentration 50 ppm, adsorbent dosage 0.2 g, contact time 60 min, solution temperature 25 °C, and pH 6 for Pb^2+^ and pH 7 for Cd^2+^. Adsorption isotherm models (Freundlich, Langmuir, Dubunin–Radushkevich, and Temkin isotherm models) as well as kinetic models (pseudo-first-order, pseudo-second-order, Elovich, and intra-particle diffusion models) were employed to evaluate the experimental results. The adsorption of Cd^2+^ and Pb^2+^ on Muc/DTPA fitted well within the Langmuir isotherm model and followed pseudo-second-order kinetics. Thermodynamics parameters of metal adsorption indicated exothermic and spontaneous processes. Results were applied to the real wastewater that showed high Cd^2+^ and Pb^2+^ removal.

## Introduction

Heavy metals removal from different water sources (natural water and ground drinking water) and wastewater have attracted considerable attention from scientists. Several methods have been modified for treating wastewater from heavy metals which include coagulation, precipitation, reduction, flotation, ion exchange, adsorption, electrochemical, membrane technology electrolysis, and membrane processes (Rashed et al., 2020; Mahdavi et al., 2013). Among these technologies, adsorption has a widely application, with high advantages of an efficient and cost-effective alternative to conventional contaminated water treatment facilities. Adsorption also does not result in the formation of harmful substances (Gürses et al., 2014; Khataee et al., 2013).

Low-cost adsorbents include natural substances such as clays, zeolites, and chitosan, and some industrial wastes (including fly ash, coal, and oxides) were used for wastewater treatment (Cheira et al., 2019; Rashed et al., 2019). One of the clay minerals in the mica family is muscovite. Natural 2M1 muscovite is a 2:1 phyllosilicate silicate mineral with the formula KAl_2_(Si_3_AlO_10_)(OH)_2_.

Muscovite is a hydrated magnesium aluminum silicate mineral resulting from iron-bearing phlogopite micas and weathering biotite. Its advantages include high availability, low-cost adsorbent, easy handling, and selectivity over other natural adsorbents (Katsou et al., 2011). The octahedral Al(O/OH)_6_ sheet and two tetrahedral SiO_4_ sheets make up the mica group minerals (Muazu et al., 2020).

As the results of the applity of their charge can change and cation exchange, layered silicates have been widely studied and applied for preparation of nanocomposite materials. It is advantageous to occupy the interlayer area while creating layered silicate nanocomposite materials. Muscovite is a layered silicate mineral that is widely used in industrial applications (absorbents, automobiles, packaging, and electrical appliances (Bao et al., 2020). Yuan etr al. (2018) synthesized 20–45 nm muscovite at 250 °C for 18–72 h using K-feldspar.

Clay minerals consist of interlayer space that has the can introduce intercalate agents, so some organic agents were intercalated into clay minerals, and the resultant intercalates exhibit fascinating properties and have numerous uses, including adsorbents, organic synthesis, polymer–clay nanocomposites, catalysis, photofunctional materials, and drug carrier in the pharmaceutical area. Several variables, including the degree of exchange, host–guest and guest–guest interactions, layer charge density, and alkyl chain length, influence the intercalation of organic compounds into clay (Lagaly et al., 2013).

The additional positive charges in the interlamellar domain are balanced by the intercalated hydrated anions. Naturally occurring anionic clays with highly organized 2D structures and superior catalytic capabilities are known as layered double hydroxides (LDHs). Enhancing the surface characteristics of the adsorbent and increasing the adsorption capacity can be accomplished by modifying the LDHs components with additional materials (Karim et al. 2022; Lan et al., 2014).

Two-dimensional (2D) nanomaterials with sheet-like structures, such as transition metal dichalcogenides (TMDs), graphene, layered double hydroxides (LDHs), metal–organic frameworks (MOFs), metal carbides and nitrides (MXenes), and covalent organic frameworks (COFs), have spurred an increased interest in adsorption and catalytic fields due to their diverse desirable properties. The narrow thickness and double-sided surface structure increased the specific surface area while also producing a significant amount of exposed active sites, which are crucial for catalytic processes. LDHs are an emerging and adaptable class of 2D inorganic layered nanomaterials, together with naturally occurring or artificial anionic clay minerals, which can be included among these 2D nanomaterials (Xie et al., 2019, Karim et al. 2022).

Various natural materials, including zeolite, natural polymers, silica, carbonaceous nanomaterials, metals, and metal oxides, were used to create nanomaterial adsorbents (Ethaib et al., 2022). Yu et al. (2006) and Yu (2007) prepared muscovite nanocomposites via the intercalation of muscovite using the inorganic–organic ion exchange steps. HafizahChe et al. (2017) prepared nanomuscovite by an alkaline salt and modified it with various concentrations of cetyltrimethylammonium bromide. El-Sheikh et al. (2020) prepared nanokaolinite photocatalyst using hydrazine hydrate as intercalate agent and used it for the removal of P-nitrophenol under UV irradiation.

Tettch et al. (2022) prepared kaolinite/muscovite composite by activation with H_2_SO_4_ and NaOH and used it to remove Pb^2+^ ions from aqueous media. Due to their distinctive properties, silica-based nanomaterials were used for adsorption of metal ions due to their highly adsorptive properties (surface area, and pore size). Ashrafi et al. (2017) prepared nanoparticle of Fe_3_O_4_@MnO_2_ MNPs with a core–shell structure and used as an effective adsorbent for removal of lead ions from aqueous solution.

The innovation of this study is to find suitable nanomaterials from abundant ores with low-cost that can be used for treatment polluted water. So, the main objective of the present study is the preparation of novel nanomoscovite from the activated muscovite by intercalation with different intercalates (DTAB-TTAB-DTPA-PA-PN). The developed adsorbents will be used for the removal of Cd and Pb ions from wastewater.

Nanosilica is an environmentally non-toxic and acceptable adsorbent. Li et al. (2019) modified silica gel with nitrilotriacetic acid and sued it for removal of Cd^2+^, Cu^2+^, and Pb^2+^ within 2 to 20 min. Kotsyuda et al. (2017) studied the removal of Cu^2+^ ions using amino-functionalized (3-aminopropyl and phenyl groups) silica nanospheres and found that the adsorption capacity increased linearly with the concentration of amino groups. Liu et al. (2018) studied Cd^2+^ ions adsorption from solutions using nano-montmorillonite and found the maximum adsorption of Cd ions was estimated to be 17.61 mg/g.

## Materials and methods

### Chemicals and reagents

A high analytical grade of these chemicals was used: lead nitrate, Pb(NO_3_)_2_, M. wt 331.21, assay (65%), Sigma-Aldrich; cadmium nitrate, Cd(NO_3_)_2_, M. wt 308.477, assay (65%), Sigma-Aldrich; TTAB (1-tetradecyl) trimethyl ammonium bromide.

(CH_3_(CH_2_)_13_N-(CH_3_)_3_Br, M.wt 336.40, assay (98%), Alfa Aesar DTAB (1-dodecyl) trimethyl ammonium bromide (CH_3_(CH_2_)_11_N(CH_3_)_3_Br, M.wt 308.35, assay (99%), Alfa Aesar DTPA diethylene tri-amine penta-acetic acid (C_14_H_23_N_3_O_10_), M.wt 393.35, assay (100%), Alfa Aesar.

## Instruments and tools

All the batch experiments were carried out in Pyrex conical beaker (100 ml) at room temperature under mechanical stirring (150 rmp); the solution pH was adjusted with (1N) NaOH or HCl. The concentrations of the metals were determined by AAS (flame atomic absorption spectrophotometry, GBC 932AA).

### Preparation of working and standard solutions

Cadmium and lead standard solution (1000 ppm) was prepared: cadmium stock solution, 2,744 g Cd (NO_3_)_2_ in 0.5 M HNO_3_,Cd^2+^ = 1000 ± 0.002 ppm; lead stock solution, 1.599 g Pb(NO_3_)_2_ in HNO_3_ 0.5 M, concentration of Pb^2+^  = 1000 ± 0.002 ppm.

### Characterization of nanomuscovite adsorbent

Morphology of nanomuscovite adsorbent was examined using SEM, XRD, and FTIR. To measure the surface area and porosity of nanomuscovite adsorbent samples, the Micromeritics Tristar 3000 (Georgia, USA) device was employed.

### Sample collection

Bulk sample (5 kg) of muscovite was collected from Southeast desert, Egypt. The samples were crushed and ground to powder with agate mortar. The silica particles were removed from muscovite samples by contacting the powdered muscovite with deionized water for one day. The suspension solution was filtered, dried at 60 °C for 24 h, and sieved to 565 μm.

### Preparation of nanomuscovite via intercalation method

Intercalated muscovite complexes were prepared using different intercalating organic agents (DTAB-TTAB-DTPA-PA-PN), and then, deintercalation process was used to have nanomuscovite particles.

#### Potassium acetate intercalated into muscovite (Mus /PA)

5 g activated muscovite (Mus) was added to a 250 mL in a plastic flask, containing 1.6157 g of potassium acetate (PA) dissolved in 100 mL deionized water. The solution was filtered and the precipitate was washed with sodium hydroxide (0.1 M) until pH 7, followed by deionized water, and dried at 100 °C for 10 h (Qian et al., 2011).

#### Potassium nitrate intercalated into muscovite (Mus /PN)

1.6645 g potassium nitrate (PN) was added to 100 mL of deionized water and mixed with 5 g of activated muscovite for 3 days at 70 °C. The solution was filtered, and the precipitate was washed with sodium hydroxide (0.1 M) until pH 7 and then repeatedly rinsed with deionized water before being dried at 100 °C for 10 h.

#### DTAB intercalated into muscovite (Mus /DTAB)

1.6645 g DTAB was added to 100 mL deionized water and mixed with 5 g of activated muscovite for 3 days at 70 °C. The solution was filtered and the precipitate was repeatedly rinsed with deionized water to remove any excess bromide anions (confirmed by silver nitrate test) and then dried at 100 °C for 10 h.

#### TTAB intercalated into muscovite (Mus /TTAB)

5 g activated muscovite was added to 100 mL deionized water containing 1.6645 g TTAB and mixed for 3 days at 70 °C. The solution was filtered and the precipitate was repeatedly rinsed with deionized water to remove any excess bromide anions (confirmed by silver nitrate test) and then dried at 100 °C for 10 h.

#### DTPA intercalated into muscovite (Mus /DTPA)

5 g activated muscovite was added to a 250-mL plastic flask, containing 6.475 g of DTPA dissolved in 100 mL deionized water. The mixture was mixed for 3 days at 70 °C and filtered and the precipitate was washed with sodium hydroxide (0.1 M) until pH 7, followed by deionized water several times and then dried at 100 °C for 10 h.

### Optimal parameters for the maximum adsorption of Pb^2+^ and Cd^2+^ by the prepared nanomuscovite

#### Solution pH effect

Solution pH effect on Pb^2+^ and Cd^2+^ removal efficiency by nanomuscovite was studied by stirring 0.1 g of nanomuscovite with 50 mL of 50 ppm Pb^2+^ and Cd^2+^ at various pH (2, 4, 5, 6, 7, 8, and 10). The mixture was filtrated by filter paper Whitman No 42, and the concentration of Pb^2+^ and Cd^2+^ in the filtrate was measured by atomic absorption spectrophotometer (AAS).

#### Adsorbent dose effect

Nanomuscovite dosage ranging from 0.05 to 1.0 g was stirred with 50 mL of 50 ppm Pb^2+^ and Cd^2+^ at pH 6 for Pb^2+^ and pH 7 for Cd^2+^. After filtration, the concentrations of Pb^2+^ and Cd^2+^ were measured using AAS.

#### Effect of initial metal concentration

0.2 g of nanomuscovite was stirred with 50 ml of Pb^2+^ and Cd^2+^ solutions with initial concentrations (10, 20, 30, 50, 75 and 100 ppm) and pH 6 for Pb^2+^ and pH 7 for Cd^2+^. The mixture was filtrated by filter paper Whitman No 42, and the metal concentration was measured using AAS.

#### Effect of contact time

0.2 g nanomuscovite was stirred with 50 mL 50 ppm Pb^2+^ and Cd^2+^ solutions at contact times (30, 60, 120 and 240 min) and pH 6 for Pb^2+^ and pH 7 for Cd^2+^. After filtration, the concentrations of Pb^2+^ and Cd^2+^ were measured using AAS.

#### Effect of temperature

0.2 g nanomuscovite was stirred with 50 mL 50 ppm Pb^2+^ and Cd^2+^ solutions for 60 min. at different temperatures (25, 35, 45, and 55 °C) keeping pH6 for Pb^2+^ and pH7 for Cd^2+^. The mixture was filtrated by filter paper Whitman No 42, and the metal concentration was measured using AAS.

## Results and discussion

### Characterization of nanomuscovite

#### XRD analysis

**Fig. 1:**
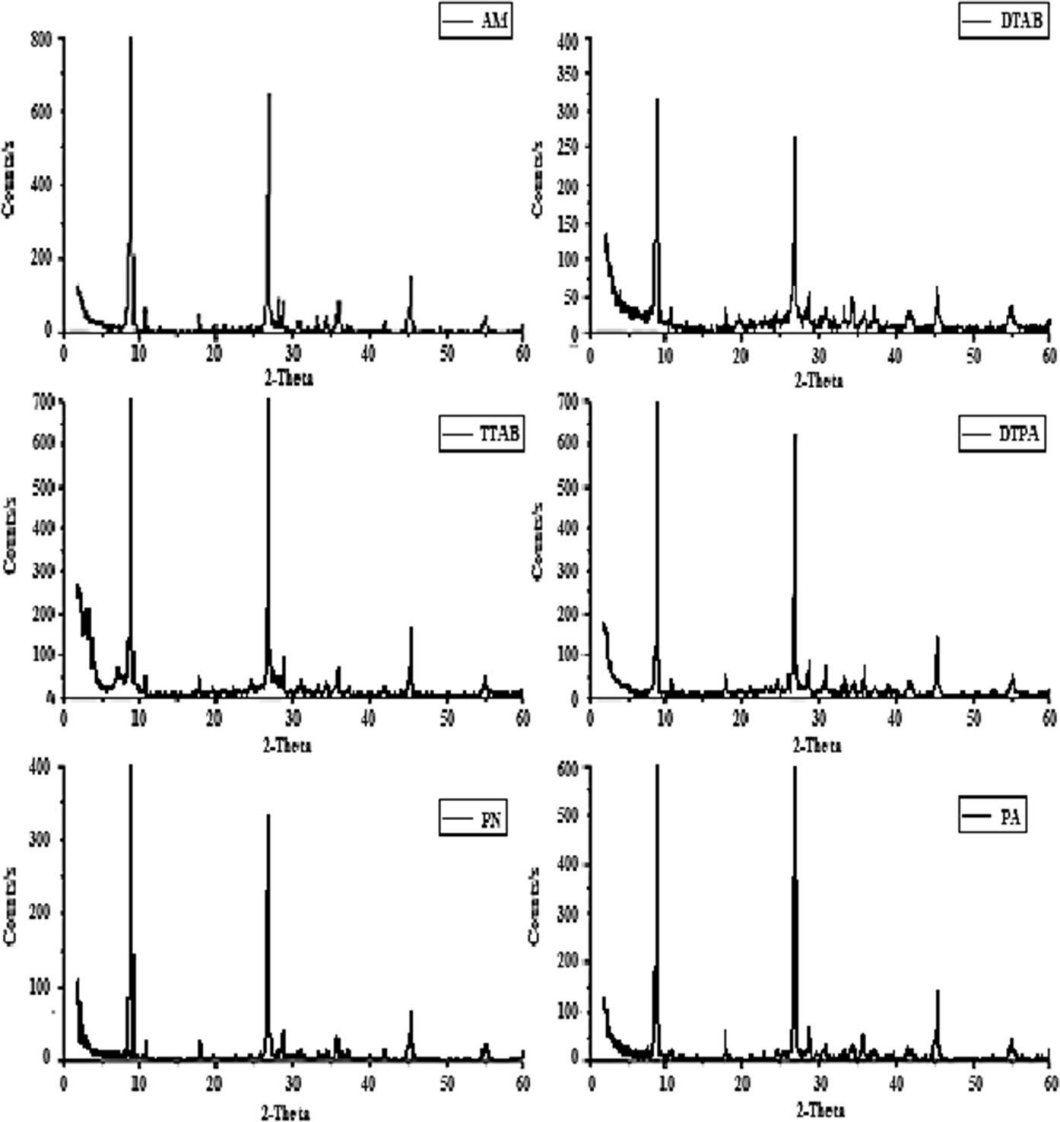
XRD of prepared nanomuscovite samples

XRD provides information about the degree of the exfoliation of the organoclay. Activated muscovite shows two intense diffraction peaks at 2θ values of 8.9° and 26.8°, less intense peaks at 2θ of 17.8°, 26.6°, 35.9°and at 2θ 45.3°. The main peak at 8.87° corresponds to the interplanar spacing of 9.98 Å, which are all associated with muscovite as determined by JCPDS data (JCPDS: 00–058-2016). Crystallite size was determined by Scherer's equation (Scherrer P. (1918) D = (Kλ)/ (βcos θ), where D is the particle diameter size, λ is the wavelength of the X-ray radiation (λ = 0.154056 nm), K is the Scherer constant (K = 0.891), θ is the characteristic angle of diffraction (2θ = 8.9º), and β is the full width at half maximum of the (001) plane after correcting with instrumental broadening using the Warren function. The XRD pattern for nanomuscovite samples prepared in the presence of DTPA, PA, PN, TTAB, and DTAB is obtained in Fig. 1. From this figure, it was noticed that the nanomuscovite samples display the same characteristic peaks as activated muscovite**,** suggesting the maintenance of the original crystallinity for the inorganic matrix when the reactions take place, where it shows two intense peaks at 8.9° and 26.8°, less intense peaks at 2θ of 17.8°, 26.6°, 35.9°, and at 2θ 45.3° as determined by JCPDS data (JCPDS: 00-058-2016). But in the case of TTAB-treated muscovite, a new form of rectorite (JCPDS: 00-014-0183) has appeared. It was observed that the crystalline size became smaller, which indicates that all intercalating agents reduced the crystallite size as summarized in Table 1. The high intensity and smallest crystallite size were obtained for the sample muscovite/DTPA.

**Table 1:** Illustration of crystallite size for nanomuscovite samples

Crystal size (nm)	2 Theta (degree)		
	8.9	26.8	45.3
Activated muscovite	70.04	68.4	53.24
Mus/TTAB	40.3	35.5	40.5
Mus/DTAB	38.05	34.4	37.05
Mus/DTPA	33.5	26.3	36.1
Mus/PA	44.3	36.3	41.3
Mus/PN	46.3	38.8	43.2

#### TEM examination

**Fig. 2:**
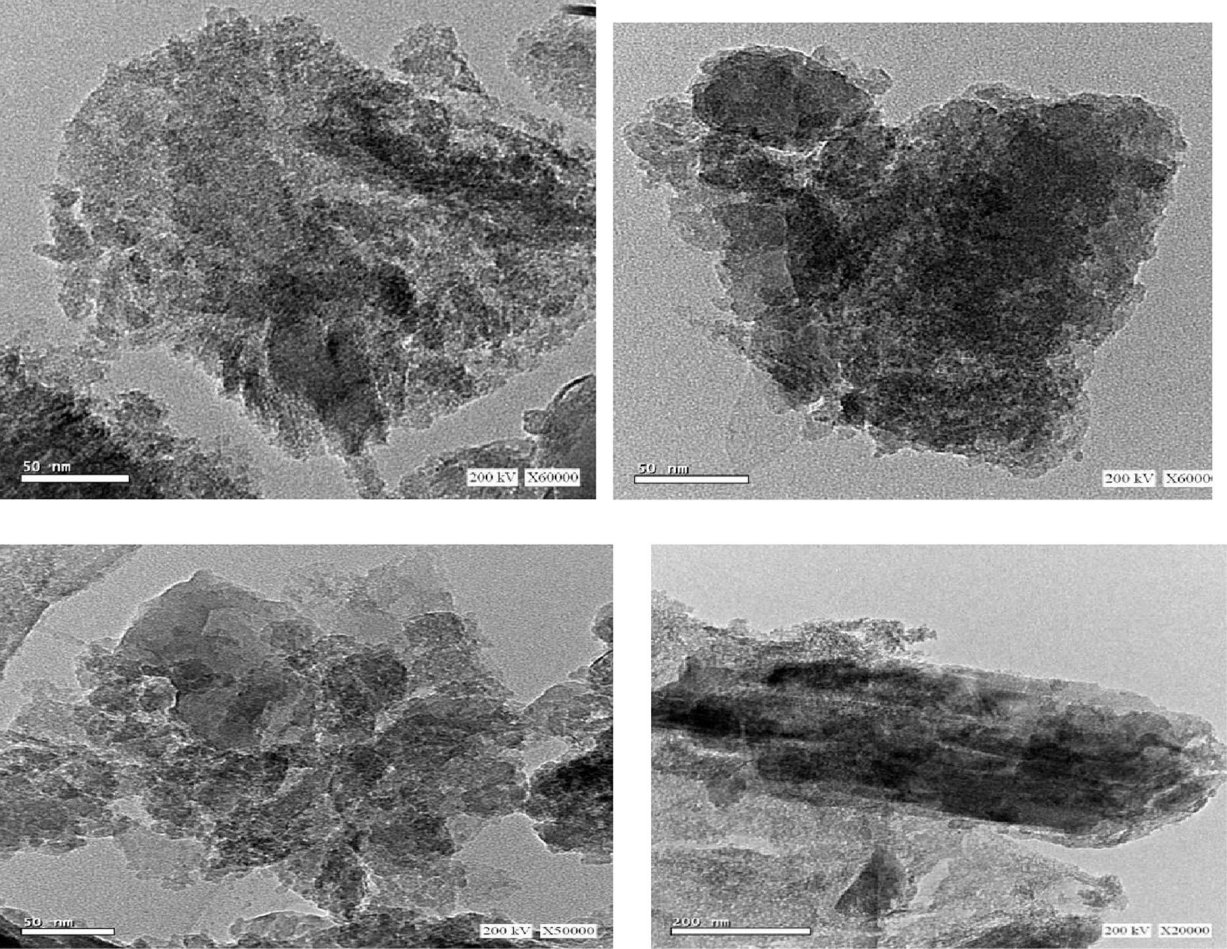
TEM of nanomuscovite

Figure 2 shows the TEM image of Mus/DTPA nanomuscovite sample. The face size of the majority of the particles is less than 100 nm, demonstrating the plate-like form of the muscovite particles and the compactness of the aggregated plates from the margins. Strong intermolecular forces cause the muscovite layers to be tightly packed together, and the smaller size of nanomuscovite particles than that of activated muscovite suggests that organic molecules may be intercalated between the muscovite layers.

**Fig. 3:**
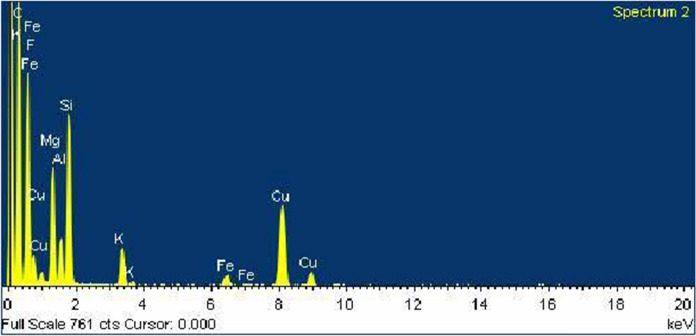
EDX data for nanomuscovite elemental compositions

From Table 2 and Fig. 3, EDX spectra reflect the muscovite purity and end to that the particle is composed of O, Mg, Si, Al, and K.

**Table 2:** EDX data for nanomuscovite elemental compositions

Element	C	O	Mg	Al	Si	Fe	K	Cu
% weight	6.67	35.36	8.48	3.15	30.37	3.27	5.1	7.5

#### FTIR analysis

**Fig. 4:**
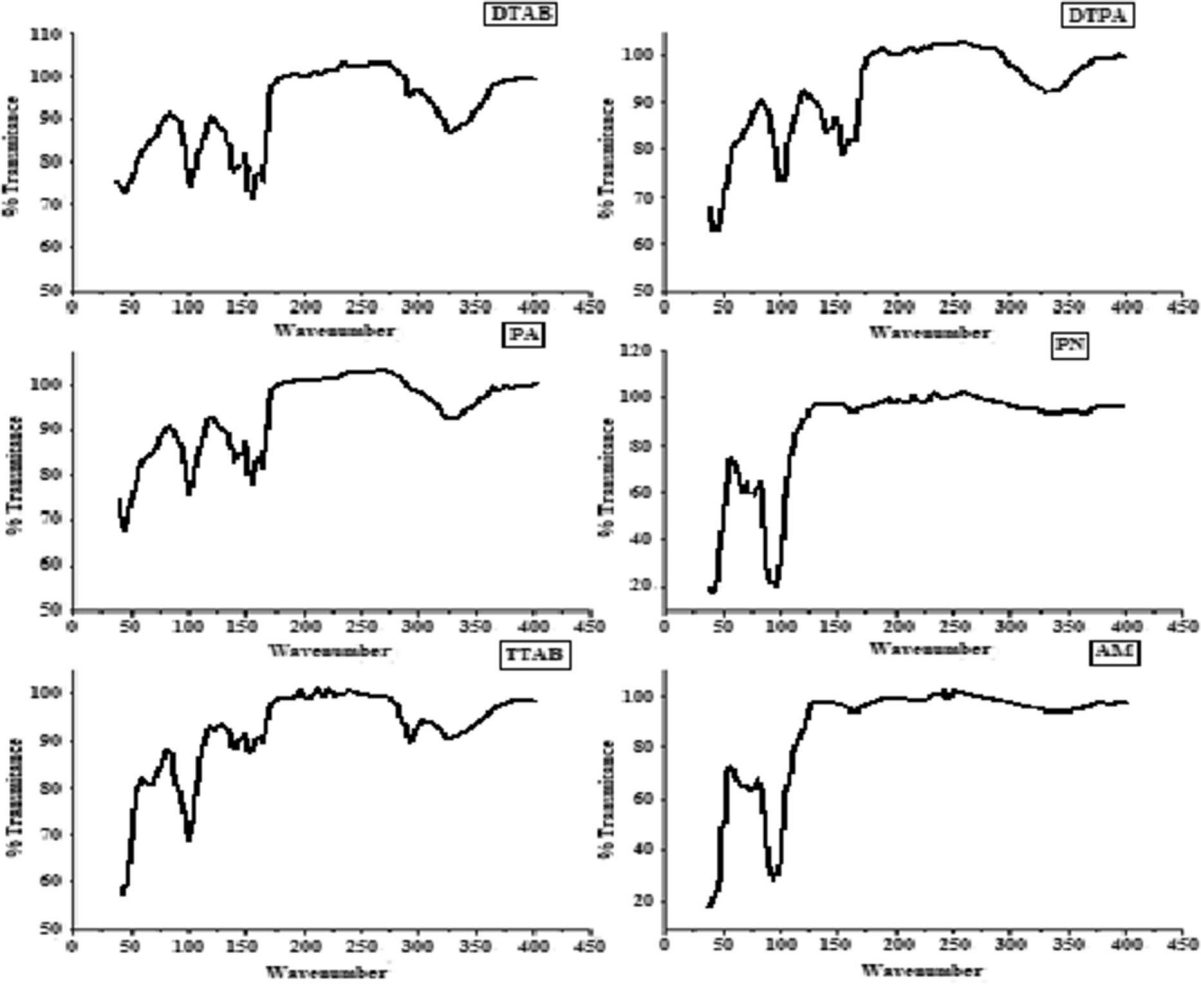
FTIR of prepared nanomuscovite samples

Figure 4 shows the FTIR spectra of all nanomuscovite samples prepared by different intercalating agents between 4000 and 400 cm^−1^. The spectrum exhibits the typical appearance, where a peak at 1016 cm^−1^ corresponds to the stretching of the Si–O bond and the bands at 2922 and 2855 cm^−1^ correspond to the stretching vibration modes of the alkyl chain from the quaternary ammonium modifier. The peak at 440 cm^−1^ corresponds to the bending of the Mg–O bond. The bands of Al–O bond bending are observed at 918 cm^−1^. The peaks at 1635 and 3298 cm^−1^ are assigned to hydration HOH and –OH vibration groups between the tetrahedral and octahedral sheets (Nooshabadi et al., 2014). The band at ∼793 cm^−1^ is for the OH bending vibrations with respect to the silicate plate in the muscovite (Chen et al., 2020). The bands at 1403 cm^−1^ and 1542 cm^−1^ are corresponding to the symmetric and asymmetric stretching vibration of the CH_3_COO- group, respectively. The 1395–1440 cm^−1^ band is attributed to the formation of coupling between C–O vibration and deformation vibration mode of the O–H groups (Ding et al., 2011). In the case of PN/muscovite, not all peaks appear and have a new band around 1385 cm^−1^ which is attributed to N–O stretching.

#### Surface area, BET, and BJH analysis

The surface area of the prepared samples was also estimated by the BET method, and it was found that surface area values of prepared samples have the following order: Muc/DTPA (195.15 m^2^g^−1^) > Muc/DTAB (190.97 m^2^g^−1^) > Muc/TTAB (182.46 m^2^g^−1^) > Muc/PA(178.45 m^2^ g^−1^) > Muc/PN (172.62 m^2^ g^−1^) > AM(137.659 m^2^ g^−1^). Table 3 shows that the pore size of Muc/DTPA was smaller one due to decreasing the particles of muscovite.

**Table 3:** Pore size and surface area of prepared nanomuscovite samples

Sample	Surface area (m^2^/g)	Pore size (nm)	Pore volume (cm^3^/g)
Activated Muscovite (AM)	137.65	14.50	0.1450
Muscovite intercalated with (DTAB)	190.97	13.94	0.1918
Muscovite intercalated with (TTAB)	182.46	14.05	0.1741
Muscovite intercalated with (DTPA)	195.15	13.82	0.210
Muscovite intercalated with (PA)	178.45	14.21	0.1521
Muscovite intercalated with (PN)	172.62	14.36	0.1348

### A comparative study of the prepared nanomuscovite samples for Cd^2+^ and Pb^2+^ removal efficiency

For comparison between the prepared nanomuscovite samples by adsorption of cadmium and lead (Table 4) it is clear that Muc/DTPA is the best one for the removal of cadmium and lead and these results have been confirmed previously. So, the Muc/DTPA will be used in all the other experiments.

**Table 4:** Removal percentage of Cd^2+^ and Pb^2+^ by prepared nanomuscovite samples

Sample	% Pb^2+^ removal	% Cd^2+^ removal
Mus/TTAB	80.3	77.4
Mus/DTAB	82.0	79.7
Mus/DTPA	85.6	82.6
Mus/PA	77.3	75.4
Mus/PN	76.0	74.7

### Optimal parameters for the adsorption of Cd^2+^ and Pb^2+^ on nanomuscovite (Muc/DTPA)

#### Effect of pH on metals adsorption

**Fig. 5:**
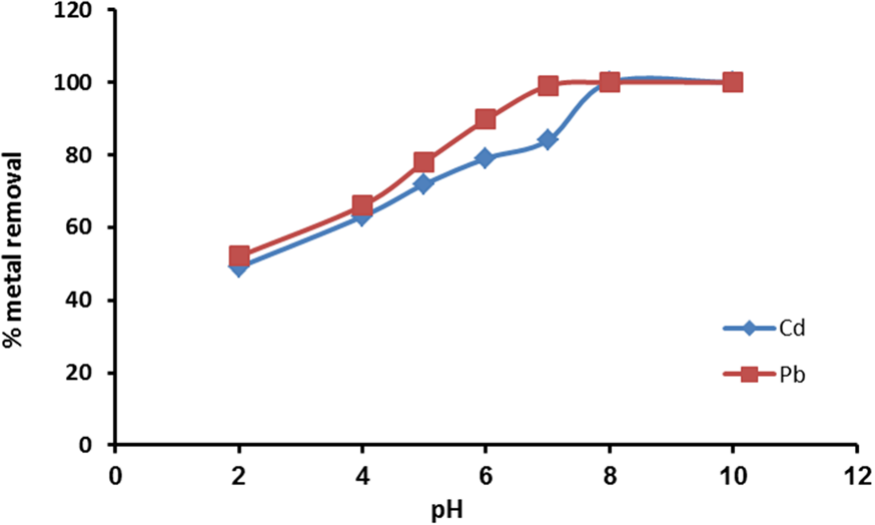
Cd^2+^ and Pb^2+^ adsorption on nanomuscovite according to pH at constant initial metal concentration 50 ppm, adsorbent dosage 0.2 g, contact time 60 min, and solution temperature 25 °C

One of the key factors affecting how metal ions adsorb is the pH of the system, which can alter the metal forms in solution and have an impact on the surface characteristics of the adsorbent in terms of the dissociation of functional groups and surface charges. The outcomes demonstrated that as the pH rose, the amount of heavy metal ions that could be removed by adsorption on nanomuscovite (Muc/DTPA) increased (Fig. 5). The adsorption removal percentage increased from 49 to 84% when the pH increased from 2 to 7 for Cd^2+^ and increased from 52 to 89.7% when the pH increased from 2 to 6 for Pb^2+^. At a low pH, the competition at the binding surfaces between heavy metal ions and H^+^ ion occurs which results in a lower repulsion of the adsorbed metal **(**Sdiri et al., 2012**)**. With the increase in solution pH, the deprotonation of the nanomuscovite surface occurs, resulting in an increase in the number of negative charge sites and so resulting in the high adsorption of metal ions on nanomuscovite **(**Zhu et al., 2013**)**.

Liu et al., (2018) found that by using nano-montmorillonite (NMMT) at pH 7, maximum Cd ions adsorption from aqueous solutions occurs. Eloussaief and Benzina (2010) investigated the efficiency of removing Pb^2+^from aqueous solutions using natural and acid-activated clays and discovered that the Pb^2+^ ions adsorption increased with pH up to a neutral pH (7.0). Yin et al. (2018) revealed that Pb(II) was effectively adsorbed in solution at pH 4 using a nanoillite/smectite clay as adsorbent. Al-a'qarbeh et al., (2020) investigated the Pb^2+^ ions adsorption from aqueous solution using the nanoplatelets client and found the maximum adsorption capacity of Pb^2+^ ions at pH 7. Using Saudi Arabian clay, Ahmad et al. (2014) investigated the removal of Pb^2+^ and discovered that pH 6 had the highest adsorption capability.

#### Effect of adsorbent dosage on heavy metal adsorption

**Fig. 6:**
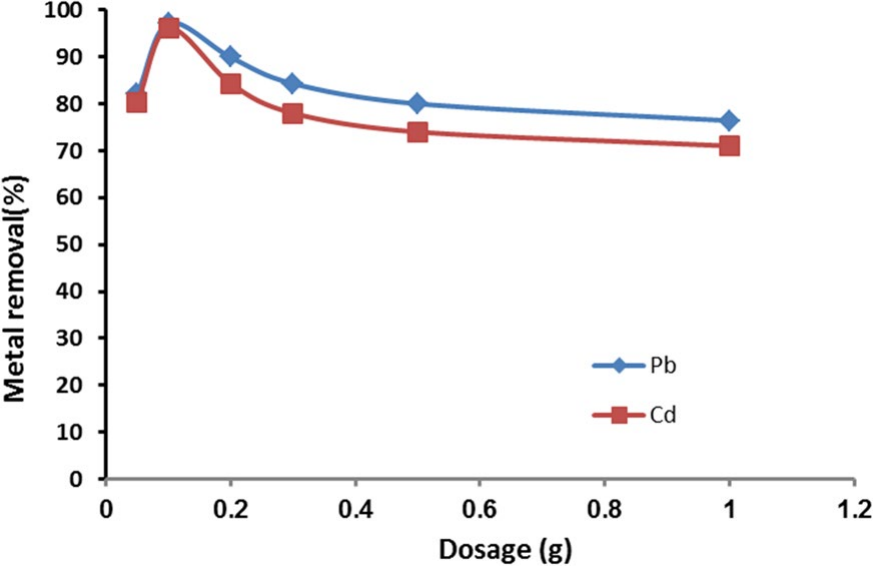
Cd^2+^ and Pb^2+^ adsorption on nanomuscovite according to adsorbent dosage at constant initial metal concentration 50 ppm, contact time 60 min, solution temperature 25 °C, and pH 6 for Pb^2+^ and pH 7 for Cd^2+^

To investigate the effect of varying nanomuscovite adsorbent doses on the uptake of Cd^2+^ and Pb^2+^ from aqueous solution, different nanomuscovite doses (0.05, 0.1, 0.2, 0.3, 0.5, and 1.0 g) were studied with constant parameters (initial metal concentration, pH, contact time, temperature). The results (Fig. 6) show that increase in adsorbent dose from 0.05 to 0.2 g increased the adsorption percentage of the metal ions from 82 to 97.1% for Pb^2+^ and from 80.2 to 96.1% for Cd^2+^; as a result of increased negative charge on the surface and the electrostatic potential decrease near the solid surface, it led to sorbent–solute interaction.

Cadmium and lead removal percentages, therefore, dropped when the effects of accumulation on the nanomuscovite adsorbent occurred with increases in its dose of more than 0.2 g, and increases in active sites with higher surface area also had little effect after equilibrium was reached **(**Gerçel & Gerçel, 2007**)**. Similar results to our study have been reported by Unuabonah et al. (2007) for the adsorption of Cd and Pb ions from solutions by adsorbent (tripolyphosphate-impregnated kaolinite clay).

#### Effect of heavy metals concentration on the adsorption

**Fig. 7:**
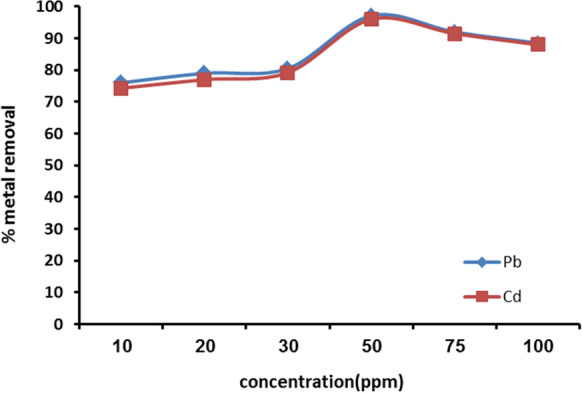
Cd^2+^ and Pb^2+^ adsorption on nanomuscovite according to initial concentration at constant adsorbent dosage 0.2 g, contact time 60 min, solution temperature 25 °C, and pH 6 for Pb^2+^ and pH 7 for Cd^2+^

The initial metal concentration effect on the removal of Cd^2+^ and Pb^2+^ by the adsorbent is represented in Fig. 7. The data evidence that the removal percentage increases from 76 to 97.1% for Pb^2+^ and from 74 to 96% for Cd^2+^, as the metal initial concentration increases from 10 to 50 ppm. This might be because Cd^2+^ and Pb^2+^ are more mobile on the surface of nanomuscovite due to the concentration differential between the adsorbate and its outer surface, increasing the driving force **(**Zhu et al., 2013**)**. After then, the metal removal efficiency decreases with increased metal concentration; the reason is that when the metal concentration rises, the active surface sites of the adsorbent become saturated, which lowers the percentage of metal removal. Additionally, additional adsorption sites are needed when Cd^2+^ and Pb^2+^ are present in the solution at greater starting concentrations **(**Masindi & Gitari, 2016**)**. The final result refers to that the best concentration of Cd^2+^ and Pb^2+^ was 50 ppm for the adsorption on nanomuscovite adsorbent.

Liu et al. (2018) studied the cadmium ions adsorption from aqueous solutions using nano-montmorillonite (NMMT) at an initial Cd ion concentration (22.4–224 mg/L) and found that the maximum Cd adsorption (17.61 mg/g) was at 22.4 mg/L Cd^2+^ concentration. David et al. (2016) prepared intercalated clay nanoparticles using cetylpyridinium chloride (CPC) and tetradecyltrimethylammonium bromide (TTAB). The resulted nanoparticles C-TTAB and C-CPC were applied to remove Cd, Pb and pentachlorophenol (PCP) through batch process. They reported the initial concentration for removal of Pb by CNP was 80 ppm and 50 ppm for Cd.

#### Effect of contact time on heavy metals adsorption

**Fig. 8:**
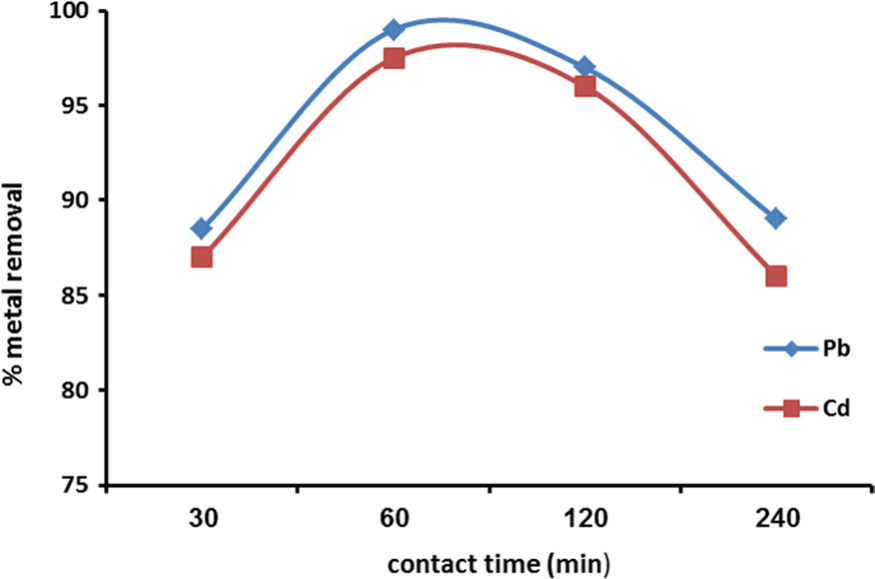
Cd^2+^ and Pb^2+^ adsorption on nanomuscovite according to contact time, at constant initial metal concentration 50 ppm, adsorbent dosage 0.2 g, solution temperature 25 °C, and pH 6 for Pb^2+^ and pH 7 for Cd^2+^

The results of contact time effects on the efficiency of Cd and Pb ion removal are presented in Fig. 8. The results revealed that the adsorption of Cd^2+^ and Pb^2+^ increased rapidly with time up to 60 min in which it reached the metal removal 95%; after then, the metal removal rate decreased as the result of the Cd^2+^ and Pb^2+^ accumulation on the vacant sites of nanomuscovite. The rapid increase in metal adsorption with time resulted a large number of vacant sites.

#### Effect of temperature on heavy metals adsorption

**Fig. 9:**
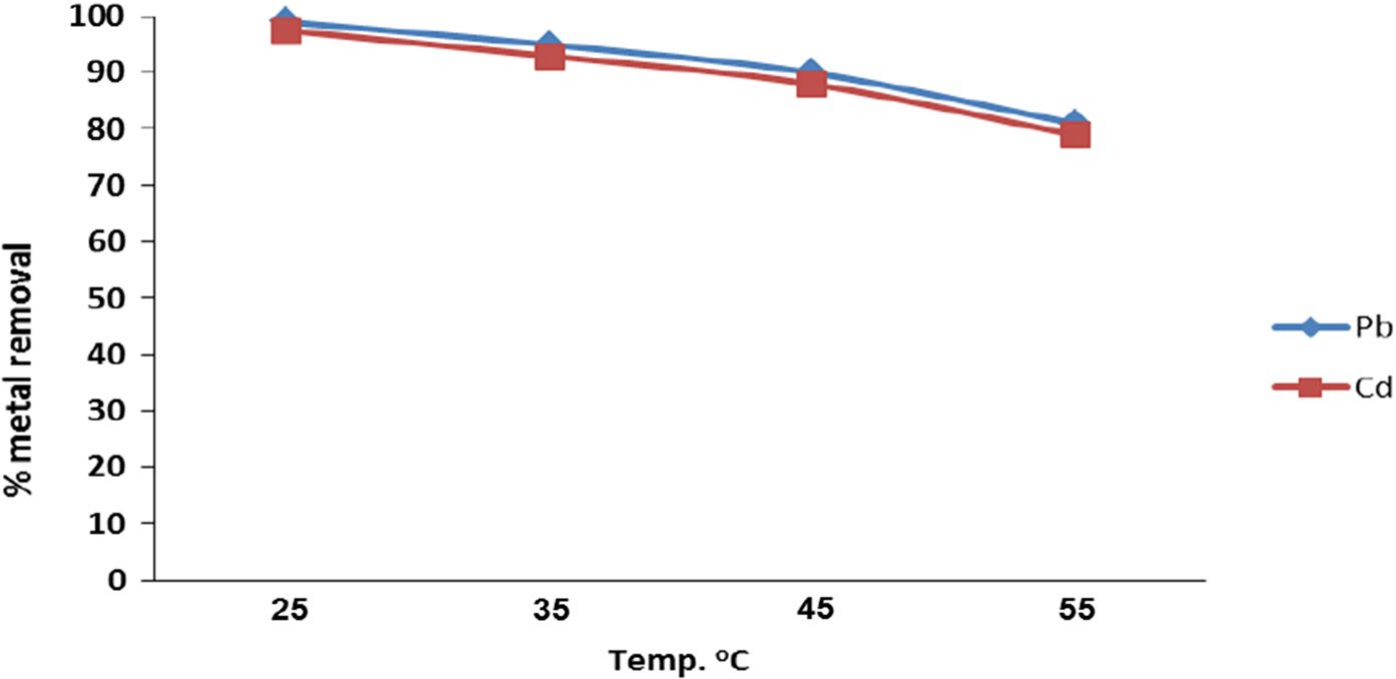
Cd^2+^ and Pb^2+^ adsorption on nanomuscovite according to temperature at constant initial metal concentration 50 ppm, adsorbent dosage 0.2 g, contact time 60 min, and pH 6 for Pb^2+^ and pH 7 for Cd^2+^

The temperature effect on the adsorption of Cd^2+^ and Pb^2+^ on nanomuscovite had been studied with different temperatures (25 °C, 35 °C, 45 °C, and 60° C). The results (Fig. 9) showed that the removal of Cd^2+^ and Pb^2+^ on nanomuscovite was high at room temperature (25 °C); after then, it decreased gradually. The adsorption effectiveness decreased from 99 to 81% for Pb and from 97.2 to 79% for Cd when the temperature was raised to 55 °C. Since the van der Waals bonds break down with increased temperature, the active sites are reduced (Negm et al., 2022**)**. Because of this, Pb and Cd ions can be better adsorbed by nanomuscovite at room temperature (25 °C) than at any other temperature.

### Adsorption isotherm study

Studies of adsorption isotherms are helpful to understand how ions move from the adsorbents during adsorption process. Characterization of Pb and Cd adsorption on the nanomuscovite approach was done using Freundlich, Langmuir, Temkin, and Dubinin–Raduskevich (D–R) isotherm models.

The experimental data ( solution temperature 25 °C, initial metals concentration 50 ppm /50 ml, 0.2 g adsorbent dosage, and 60 min contact time) are presented in Table 5.

**Table 5:** Isotherm data for Pb^2+^ adsorption on nanomuscovite according to initial metal concentration variation at 25 °C

Conc. ppm	% Lead adsorbed	*C*_e_	*q*_e_	*C*_e_/*q*_e_	Log *C*_e_	Log *q*_e_	E2	Ln *C*_e_	Ln *q*_e_	1/*C*_e_
10	76	2.4	1.90	0.380	0.279	0.279	7597	0.875	0.642	0.417
20	79	4.2	3.95	0.623	0.597	0.597	2856	1.435	1.374	0.238
30	80.5	5.85	6.03	0.767	0.781	0.781	1559	1.766	1.798	0.171
50	97.1	1.45	12.13	0.161	1.084	1.08	1722	0.372	2.496	0.69
75	92	6	17.25	0.778	1.237	1.237	1488	1.792	2.848	0.167
100	88.5	11.5	22.12	1.061	1.345	1.345	4351	2.442	3.097	0.087

#### Langmuir isotherm

Langmuir isotherm model described the saturated monolayer adsorption on the homogenous surface at constant energy. Langmuir equation can be quantified by equation:

1 $$C_{{\text{e}}} /q_{{\text{e}}} = { 1}/Q_{{\text{o}}} b \, + \, C_{{\text{e}}} /Q_{{\text{o}}}$$

*C*_e_ is the equilibrium concentration of heavy metals (mg/L), *Q*_o,_ the adsorbent monolayer adsorption capacity of (mg/g), *q*_e,_ the metal equilibrium capacity at adsorbent (mg/g), and b, the Langmuir binding energy coefficient (L/mg).

Calculating the Q_o_ and b1 from Eq. 1 requires graphing *C*_e_/*q*_e_ against Ce and determining the intercept and slope (Table 6).

**Table 6:** Isotherm data for Cd^2+^adsorption on nanomuscovite according to initial metal concentration variation at 25 °C

Conc.ppm	%Cadmium adsorbed	*C*_e_	*q*_e_	*C*_e_/*q*_e_	Log *C*_e_	Log *q*_e_	E2	Ln *C*_e_	Ln *q*_e_	1/*C*_e_
10	74.3	2.57	1.858	1.384	0.41	0.269	6764	0.944	0.62	0.389
20	77	4.6	3.85	1.195	0.663	0.585	2423	1.526	1.35	0.217
30	79.2	6	5.94	1.051	0.795	0.774	1383	1.831	1.78	0.16
50	96	2	12	0.167	0.301	1.079	1029	0.693	2.48	0.5
75	91.5	6.37	17.15	0.372	0.804	1.234	1329	1.852	2.84	0.157
100	88	12	22	0.545	1.079	1.342	4012	2.485	3.09	0.083

From the data in Table 7, the correlation coefficients (*R*^2^) of Cd^2+^ and Pb^2+^ were found as 0.986 and 0.982 for nanomuscovite adsorbent, respectively, indicating that the adsorption mechanism of Cd^2+^ and Pb^2+^ ions onto the nanomuscovite surface is carried out via monolayer. The maximum monolayer adsorption capacities (*Q*_o_) of Cd^2+^ and Pb^2+^ were found to be 26.6 and 25.2 mg g^−1^, respectively, and close to the experimental capacities.

**Table 7:** Isotherms parameters for Cd^2+^ and Pb^2+^ adsorption on nanomuscovite

Parameters	Lead	Cadmium
*Langmuir isotherm model*		
Q_0_ (mg/g)	25.2	26.6
bL (L/mg)	0.498	0.35
*R*^2^	0.98	0.99
*Freundlich isotherm model*		
1/n	0.26	0.36
Kf (mg^−1^/n L^1/n^ g^−1^)	2.29	2.09
*R*^2^	0.77	0.805
*Temkin isotherm model*		
B (j/mol)	4.62	5.44
bT(j/mol)	541	460
Kt (L/g)	8.9	4.3
*R*^2^	0.97	0.98
*Dubinin–Raduskevich isotherm model*		
qD (mg/g)	7.4	7.1
E (kj/mol)	0.5	0.32
*R*^2^	0.96	0.96
*β* (mol/kJ)	2*10–6	5*10–6

Also, from the results b_L_ < 1 for the studied metal adsorption reveals that the adsorption of Cd^2+^ and Pb^2+^ ions on nanomuscovite is favorable according to Langmuir isotherm. So, the Langmuir model best fits the experimental data.

#### Freundlich isotherm

The Freundlich isotherm is applied to determine the applicability of heterogeneous surface energy in the adsorption process. The convenient formula for Freundlich is:

2 $${\text{Log }}q_{{\text{e}}} = {\text{ log }}K_{{\text{f}}} + { 1}/n{\text{ log }}C_{{\text{e}}}$$

The constants *K*_f,_ and n are the adsorption capacity and the adsorption intensity, respectively. So, when 1/n values are < 1, it indicates normal adsorption with a strong interaction between adsorbent and the metal, while if 1/*n* < 1, it indicates cooperation adsorption.

From Eq. 2, the intercept and slope of the linear plot of log qe against log *C*_e_ can be used to compute the *K*_f_ and *n*.

From Eq. 2, the *K*_f_ and n can be calculated from the intercept and slope of the linear plot of log q_e_ against log C_e_ and are tabulated in Table 7. The results obtained of *K*_f_ and 1/*n* were 0.36 and 2.09 for Cd^2+^ and 0.26 and 2.29 for Pb^2+^, respectively. The resulted 1/n values indicate that the adsorption of Cd and Pb ions onto nanomuscovite was favorable at studied conditions. The Freundlich isotherm model did not provide a better fitting than the Langmuir isotherm, as indicated by the lower correlation coefficient (*R*^2^) values (0.805 and 0.773 for Cd^2+^ and Pb^2+^, respectively) than that in the Langmuir model.

#### The Temkin isotherm

The Temkin isotherm model suggests that the population of surface binding adsorption will have a uniform distribution of binding energies.

The Temkin equation's linear form is written as:

3 $$q_{{\text{e}}} = \, B{\text{ ln }}K \, + \, B{\text{ ln }}C_{{\text{e}}}$$

*B* = RT/*b*, where b is the heat of sorption-related Temkin constant. A plot of qe versus ln Ce allows one to estimate the constants B, which is related to the heat of sorption (J/mol), and K, which is the Temkin isotherm equilibrium binding constant.

The values *K*_T_ = 4.3 and 8.9 L g^−1^, *R*^2^ = 0.98 and 0.96, and *B* = 5.44 and 4.62 J mol^−1^ for Cd^2+^and Pb^2+^, respectively, were estimated from the Temkin plot (Table 7). The heat of sorption (b_T_) indicates a physical adsorption process as the values of b_T_ are less than 8 kJ/mol. This means that the adsorption of cadmium and lead on nanomuscovite follows the Temkin model.

#### Dubinin–Radushkevich isotherm model

On a heterogeneous surface, the Dubinin–Radushkevich isotherm is typically used to express the adsorption mechanism with Gaussian energy distribution. It is used to differentiate between metal adsorption which is physical and chemical.

The linearized D-R equation:

4 $${\text{Ln }}q_{{\text{e}}} = {\text{ ln }}q_{{\text{m}}} {-}{\text{ BE}}^{{2}}$$

By graphing ln *q*_e_ versus *E*^2^ using Eq. 4, it can calculate the *β* values from the slope and q_m_ from the intercept.

Information regarding chemical and physical adsorption is provided by the mean adsorption energy (*E*). The obtained values of the mean free energy (*E*) for cadmium and lead, respectively, are 0.32 kJ/mol and 0.5 kJ/mol, respectively (Table 7).

E value for Cd^2+^ and Pb^2+^ is less than the 8–16 kJ /mol range of the adsorption reaction. Therefore, physical adsorption was used to describe adsorption of Cd^2+^ and Pb^2+^ on nanomuscovite.

From Table 7, the adsorption of Cd^2+^ and Pb^2+^ on nanomuscovite fitted well Langmuir isotherm due to the high correlation factor (R^2^) than the other isotherm models.

The R^2^ values of Cd^2+^ and Pb^2+^ using the evaluated isotherms confirm the following sequence Langmuir (best fit) > Temkin > Dubinin–Radushkevich > Freundlich.

#### Adsorption kinetic studies

Kinetic models have been proposed to identify the adsorption process' mechanism. These models offer important data that can improve adsorption effectiveness and process scale viability (Eftekhari et al., 2010). The pseudo-first-order, pseudo-second-order, Elovich models, and intra-particle diffusion were applied to examine the adsorption kinetics that characterize the effectiveness of Pb and Cd adsorption on nanomuscovite. All adsorption steps, including external film diffusion, internal particle diffusion, and adsorption, are included in these models. Tables 8 and 9 show the kinetic information for Pb and Cd adsorption on nanomuscovite.

**Table 8:** Kinetic effect of contact time and initial metal concentration (50 ppm) on the adsorption of lead by nanomuscovite at 25 °C

Time (*t*) min	% of removal	*C*_e_	*q*_t_	*q*_e− _*q*_t_	Log (*q*_e− _*q*_t_)	*t*/*q*_t_	Ln *t*	*t*^1/2^
30	99	5.75	11.063	1.538	0.19	2.71	3.4	5.48
60	95	0.5	12.375	0.225	0.65	4.85	4.09	7.75
120	90	1.5	12.125	0.475	0.32	9.9	4.79	10.95
240	81	5.5	11.125	1.475	0.17	21.6	5.48	15.49

**Table 9:** Kinetic effect of contact time and initial metal concentration (50 ppm) on the adsorption of cadmium by nanomuscovite at 25°

Time (*t*) min	% of removal	*C*_e_	*q*_t_	*q*_e− _*q*_t_	Log (*q*_e− _*q*_t_)	*t*/*q*_t_	Ln *t*	*t*^1/2^
30	87	6.5	10.875	1.325	0.122	2.76	3.4	5.48
60	97.5	1.25	12.188	0.0125	1.903	4.92	4.09	7.75
120	96	2	12	0.2	0.699	10	4.79	10.95
240	86	7	10.75	1.45	0.161	22.3	5.48	15.49

##### Pseudo-first-order model

The following is a representation of the pseudo-first-order kinetic model:

5 $${\text{Log }}\left( {q_{{\text{e}}} {-}q_{{\text{t}}} } \right) \, = {\text{ log }}q_{{\text{e}}} {-} \, \left( {K_{{1}} /{2}.{3}0{3}} \right) \, t$$

*K*_1_ is the rate constant of the pseudo-first order and q_e_ and q_t_ (mg/g) are the adsorption capacities at equilibrium and time (min), respectively.

The pseudo-first-order kinetic constants for the adsorption of Cd^2+^ and Pb^2+^ on nanomuscovite were calculated from Eq. 5 using the slope and intercept of the linear relation between time (*t*) and Log (*q*_e_ –*q*_t_). The correlation coefficient (*R*^2^) values were 0.843 and 0.854 for Cd^2+^ and Pb^2+^, respectively. The k_1_values were 0.02 and 5.8 × 10^−3^ min^−1^ for Cd^2+^ and Pb^2+^, respectively (Table 10). The pseudo-first-order model's R^2^ value for Cd^2+^ and Pb^2+^ is lower than the R^2^ values in the other models, indicating that the model is irrelevant. This indicates that pseudo-first-order equation might not be adequate to construe the mechanism of Cd^2+^ and Pb^2+^ onto nanomuscovite interactions.

**Table 10:** Calculated kinetic parameters for Cd^2+^ and Pb^2+^ adsorption on nanomuscovite

Parameters	Pb^2+^	Cd^2+^
*Pseudo-first-order*		
*K*_1_ (min)^−1^	5.8*10^− 3^	0.02
*q*_e_ (mg/g)	2.07	8.76
*R*^2^	0.85	0.84
*Pseudo-second-order*		
*K*_2_ (g/mg min)	0.018	0.015
*q*_e_(mg/g) (calculated)	12.1	12
*q*_e_(mg/g) (experimental)	11.03	11.6
*R*^2^	0.997	0.996
*Elovich model*		
α (mg/min)	2.4	2.1
*β* (g/mg)	1.3	1.1
*R*^2^	0.78	0.86
*Intraparticle diffusion model*		
*K*_i_	0.17	0.19
*C* (mg g^− 1^)	10.4	10.2
*R*^2^	0.88	0.83

##### Pseudo-second-order model

It is confirmed that the pseudo-second-order model is the rate-limiting stage in the formation of the van der Walls bond between Cd^2+^ and Pb^2+^ via electron sharing or exchange. The following is a representation of the pseudo-second-order kinetic model:

6 $$t/q_{{\text{t}}} = { 1}/K_{{2}} q_{{\text{e}}}^{{2}} + { 1}/q_{{\text{e}}} t$$

K_2_ is the rate constant of pseudo-second-order adsorption (g/mg/ min).

From Eq. 6, the plots of *t*/*q*_t_ vs time (*t*) for the adsorption of Cd^2+^ and Pb^2+^ are derived using the pseudo-second-order model. Two parameters (*q*_e_ and K_2_) were determined using the intercept and slope, respectively. High correlation coefficient (*R*^2^) values were obtained for Cd^2+^ and Pb^2+^ ions (0.996 and 0.997, respectively) (Table 10). For Cd^2+^ and Pb^2+^, the calculated equilibrium adsorption capacity (*q*_e_) values were 12 and 12.1 mgg^−1^, respectively. The adsorption of Cd^2+^ and Pb^2+^ followed pseudo-second-order model, as seen by the high conformity between the *q*_e_, cal and *q*_e_ exp values.

##### Intra-particle diffusion model

The solute transfer for solid–liquid adsorption is often characterized by an intra-particle diffusion model, which was used to determine the adsorption mechanism:

7 $$q_{{\text{t}}} = \, K_{{\text{i}}} t \, \raise.5ex\hbox{$\scriptstyle 1$}\kern-.1em/ \kern-.15em\lower.25ex\hbox{$\scriptstyle 2$} \, + \, C$$

where *C* (mgg^−1^) indicates the boundary layer effect and *K*_i_ (mg g^−1^ min^−1/2^) is the rate constant of the intra-particle diffusion model.

**Fig. 10:**
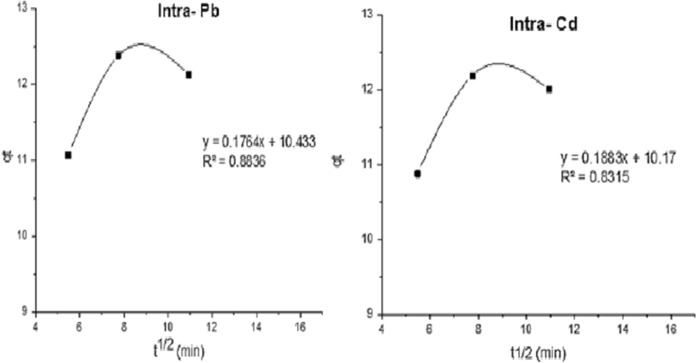
Intra-particle kinetic for Pb and Cd adsorption on nanomuscovite

The slop and intercept of the linear plot of *q*_t_ versus *t*^1/2^ allow for the determination of the *K*_i_ and *C* from Eq. 7 (Fig. 10). Table 10 contains the estimated and collected intraparticle diffusion constants for adsorption of Cd^2+^ and Pb^2+^ onto nanomuscovite.

The multilinearity forms shown in Fig. 10 indicate that there may be two or more additional steps. The first shows that the line's sharper portion can be attributable to the boundary stratum diffusion of solutions to the diffusion of divalent ions (Cd^2+^ and Pb^2+^) from the solution to the surface of nanomuscovite. The progressive adsorption stage is described in the second section, with intraparticle diffusion serving as the rate-limiting step **(**Bany-Aiesh et al., 2015; Dawodu & Akpomie, 2014; Srivastava & Hasan, 2011**)**. *K*_id_ values for Cd^2+^ and Pb^2+^ were 0.19 and 0.17 mg g^−1^ min^−1/2^, while *R*^2^ equal 0.83 and 0.88, respectively. *C* (10.2 and 10.4 mgg^−1^ for Cd^2+^ and Pb^2+^, respectively) values provide information regarding the boundary layer's thickness. The impact of the boundary strata increases with increasing intercept **(**Bany-Aiesh et al., 2015**)**. The variation in the rate of mass transfer at the first and final stages of adsorption may cause the straight lines' aberration from the origin. Furthermore, pore dispersion is not the only rate-controlling step indicated by such a divergence of the straight lines from the origin (Srivastava & Hasan, 2011**)**. Furthermore, there may be two distinct stages involved in the adsorption of two divalent ions onto nanomuscovite. The surface adsorption or rapid external diffusion stage was considered to be the first linear fraction. The piecemeal adsorption process, which is managed by intra-particle-diffusion, forms the basis of the second linear portion **(**Dawodu & Akpomie, 2014). Due to low *R*^2^ values, the sorption of Cd^2+^ and Pb^2+^ on nanomuscovite did not follow the intraparticle diffusion equation.

##### Elovich model

Elovich equation is one of the best models for characterizing these activated chemisorptions and described as:

8 $$q_{{\text{t}}} = { 1}/\beta {\text{ ln}}\left[ {\alpha \beta } \right] \, + { 1}/\beta {\text{ lnt}}$$

α is the initial adsorption, and *β*, the adsorption coefficient

In Eq. 8, when qe is plotted against ln (*t*), graphs are created from which the constants *α*, *β* and correlation coefficient (*R*^2^) are computed (Table 10). The rate-controlling step (RCS) of the Elovich model is the chemical reaction of Cd^2+^ and Pb^2+^ with the adsorbent surface, and the removal process involves multilayer adsorption **(**Hosseini-Bandegharaei et al., 2016**)**.

The results reveal that the initial Cd^2+^ and Pb^2+^ adsorption rates onto nanomuscovite were studied, and the Elovich values (*α*) were determined to be 2.1 and 2.4 mg min^−1^ Cd^2+^ and Pb^2+^, respectively. The values of β, which represent the number of adsorption sites, were 1.1 and 0.1.3 for Cd^2+^ and Pb^2+^, respectively. The adsorption of Cd^2+^ and Pb^2+^ onto nanomuscovite did not follow the Elovich equation because of low *R*^2^ values.

Last but not least, Table 10 shows that all four kinetic models used for Cd^2+^ and Pb^2+^ adsorption on nanomuscovite follow a pseudo-second-order kinetic model.

#### Thermodynamics of adsorption

The actual indications for particle applications are thermodynamic variables such (Δ*G*°), entropy (Δ*S*°), and enthalpy (Δ*H*°). Thermodynamics of adsorption was assessed in relation to various temperatures (298, 313, and 333 K).

The following equation (Ghosal & Gupta, 2017) was used to compute the thermodynamic parameters:

9 $${\text{Ln }}k \, = \, \Delta S^{{\text{o}}} /{\text{R}} - \Delta H^{{\text{o}}} /{\text{RT}}$$

*R* is temperature (K) and *T* is the gas constant (8.314 J/mol K).Table 11 lists the estimated thermodynamic parameters.

**Table 11:** Thermodynamical parameters for the adsorption of Cd^2+^ and Pb^2+^

Parameters		Cd	Pb
ΔH°, kJ/mol		− 6.2	− 8.3
ΔS°, kJ/ mol K		− 0.19	− 0.254
ΔG°, kJ/mol	298 k	− 5.4	− 7.5
	308 k	− 3.5	− 5
	318 k	− 1.6	− 2.4
	328 k	− 0.3	− 0.112
R^2^		0.987	0.965

The findings demonstrate that the characteristic sign of the Δ*G*° values was negative at all temperatures, demonstrating the spontaneous nature of these adsorptions. Additionally, it was found that the Δ*G*° values drop as *T* rises, suggesting that there is less driving force for adsorption at higher *T*. The negative sign of Δ*H*° indicates that the adsorption processes are exothermic. The results show the Δ*H*° values for adsorption of Cd^2+^ and Pb^2+^ ions are smaller than 20.9 kJ/mol which implies that Cd^2+^ and Pb^2+^ ions are physically adsorbing to nanomuscovite. The ΔH° value in the range of 2.1–20.9 kJ/mol indicates physical adsorption and 80–200 kJ/mol chemical adsorption processes. The organization of the adsorbed ions becomes less random, and the negative Δ*S*° values reflect a drop in ion concentration at the solid solution interface, indicating an increase in divalent ions adsorption on the nanocrosslinked solid phase.

## Conclusion

In this study, effective nanomuscovite adsorbent was prepared by intercalation muscovite with intercalates (DTAB-TTAB-DTPA-PA-PN).

The results of the batch adsorption of Pb and Cd on the nanomuscovite revealed high adsorption dependent on pH, contact time, adsorbent dose, initial metal concentration, and solution temperature. The adsorption kinetics of lead and cadmium ions followed the pseudo-second-order kinetic model.

In addition, the adsorption isotherm was analyzed by Langmuir, Freundlich, Dubinin, and Temkin isotherm models. The Langmuir model was fitted well to the well-explained Pb and Cd adsorption on the nanomuscovite with maximum adsorption 25.2 mg/g for Pb and 26.6 mg/g for Cd. The thermodynamic parameters values such as Δ*G*°, Δ*H*°, and Δ*S*° were calculated for the nanomuscovite adsorbent and ended that the Δ*G*° value was negative, indicating the adsorption was spontaneous.

The easy-to-use, environmentally safe, and effective procedure of this adsorbent would offer a crucial technique for removing industrial wastewater.

## Data Availability

Not applicable.
